# A closer look at ‘Cheap White’ cigarettes

**DOI:** 10.1136/tobaccocontrol-2015-052540

**Published:** 2015-09-28

**Authors:** Hana Ross, Nicole Vellios, Katherine Clegg Smith, Jacqueline Ferguson, Joanna E Cohen

**Affiliations:** 1 School of Economics, University of Cape Town, Cape Town, Western Cape, South Africa; 2 Johns Hopkins Bloomberg School of Public Health, Baltimore, Maryland, USA

**Keywords:** Illegal tobacco products, Price, Taxation, Tobacco industry

## Abstract

**Background:**

Given the prominence of Cheap Whites in illicit tobacco discussions, we examined various definitions, market presence, brand proliferation, manufacturers, production locations, trademark ownership, prices and compliance with tax stamp and warning labels.

**Methods:**

Data from peer-reviewed and grey literature, newspapers, trademark registries, governments/international organisation reports, and the tobacco industry were contrasted with two visual legal requirements (tax stamps and warning labels) and prices from the Tobacco Pack Surveillance System (TPackSS).

**Results:**

Multiple sources identified 82 Cheap White brands and 53 manufacturers operating at least 82 production facilities. One-third of these manufacturers are in the free zones of Russia, Cyprus and the UAE. Two-thirds of the 37 Cheap White brands in the TPackSS had neither the correct health warning nor the required tax stamp in at least one country where they were purchased. Cheap Whites are on average less expensive than all other brands, but the price gap is often not as large as anecdotally reported. The cheapest Cheap White cigarettes purchased in one of the TPackSS countries irrespective of the presence of legal signs were still more expensive than the least expensive other brands satisfying both legal requirements.

**Conclusions:**

We confirmed that many Cheap White brands do not comply with the legal requirements in countries where they are sold, but also found that some of these cigarettes appear to be sold legally even outside their country of origin. The presence of untaxed Cheap Whites undermines tobacco tax policies, while the availability of legal cheap cigarettes is a public health concern.

## Introduction

Cheap Whites, also known as Illicit Whites, have been described as cigarettes manufactured by legitimate business enterprises with a large share of the production being sold without all applicable duties paid, usually outside the jurisdiction where they are produced.

Although the terms ‘Illicit Whites’ and ‘Cheap Whites’ are used interchangeably in the literature, we use the term ‘Cheap Whites’, because we determined that some of these brands are sold legally.

Cheap Whites have emerged in the illicit trade channels over the past decade and several sources indicate their growing importance on illicit markets.^1–3^ Until now, the issue of Cheap Whites has been primarily analysed by Transnational Tobacco Companies (TTCs),^4–7^ because it is in their interest to draw attention to illicit trade in which other companies are involved. Within this context, an independent examination of this phenomenon is valuable.

On the illegal market, the advantage of Cheap Whites over counterfeited cigarettes is that they are not subject to legal action regarding trademarks. This lower risk translates into lower costs, which may allow the manufacturers of Cheap Whites to invest in more expensive machinery and higher quality material without risk of confiscation. As a result, the quality of Cheap Whites in terms of ‘smoothness’, flavour and packaging is usually better than that of counterfeits, and even motivates their counterfeiting.^2^ This increases the competitive advantage of Cheap Whites on the market where they directly compete with TTC brands.^2^

The distribution of Cheap Whites closely resembles the distribution of legal cigarettes, because the sale to the first purchaser is usually legal. Some Cheap Whites then make their way to the illicit market via the subsequent purchasers, often using elaborate routings, which makes it difficult to trace the source and the country of origin.^2^ ^8^

We identified only three academic articles that touched briefly on the issue of Cheap Whites. Lo *et al*^9^ report the occurrence of Cheap White brands in Taiwan as early as 2007, while Joossens and Raw^3^ describe the appearance of Cheap Whites in Europe in 2008 and identify them as an emerging source of illicit cigarettes. Gilmore *et al*^10^ reported that TTCs focus heavily on the presence of Cheap Whites on the illicit cigarette market while ignoring the fact that the majority of this market is still dominated by TTC brands.

This study aims to fill the void in the literature by summarising the existing evidence and analysing new data on Cheap Whites to examine how these cigarettes are defined, their market presence, brand proliferation, sources, trademarks, compliance with local tax and warning labels laws, and their prices.

## Methods

Information on Cheap White brands, their market presence and their production facilities were obtained via a review of the published scientific and grey literature, online news articles, trademark registries, and documents published by various international organisations, governments and the tobacco industry.

The World Customs Organization (WCO) annual reports on illegal trade covering tobacco products were particularly useful, even though only partial 2008–2011 reports and full 2012–2013 reports are publicly available.^2^ ^11–16^ The WCO data are primarily based on seizures in Europe. We also consulted publications of the European Commission (EC),^17–19^ Office de Lutte Anti-Fraude (OLAF, the European Anti-Fraud Office),^20^ Europol,^21^ Interpol,^22^ the United Nations,^23^ and online sources of Custom Departments and agencies in charge of enforcement in the UK,^24^ Germany,^8^ Poland^25^ and Malaysia.^26^ Further, we reviewed resources provided by the International Consortium of Investigative Journalists and information published in news articles^26–28^ to cross-verify information from other sources.

Reports commissioned by tobacco companies included the ‘Project Star’^5^ ^6^ ^29^ and ‘Project Sun’^7^ reports that analyse illicit cigarette consumption in the European Union (EU), the Asia-11^4^ and the Asia-14^30^ reports that focus on illicit cigarette consumption in Asia, as well as reports of the UK Tobacco Manufacturers’ Association (TMA)^31^ and the International Tax and Investment Centre.^32^

For the purpose of this report, we rely on international organisations, governments and the tobacco industry to identify brands as either Cheap Whites or Illicit Whites. Therefore, non-Cheap Whites are brands that have not been identified as Cheap Whites or Illicit Whites in any of the data sources described above. These can also include brands of major tobacco companies (sold either with or without all required taxes paid) and counterfeit products.

Information on the manufacturer, prices and compliance with local tax stamp and warning labels for 14 low income and middle income countries across five of the six WHO regions was obtained from the Tobacco Pack Surveillance System (TPackSS), a database developed by the Institute for Global Tobacco Control at the Johns Hopkins Bloomberg School of Public Health. TPackSS data were generated through a 2013 systematic purchase of cigarette packs and consist of images of cigarette packs, and data on price, place of purchase, manufacturer, presence of tax stamp and health warnings, among others.^33^ The purpose of the data collection was not to study Cheap Whites, but as it includes all brands available for purchase via a broad sample of vendors in three diverse cities in each country (and 5 cities in China), it captured some of them. Two features of a pack determined whether it was licit or illicit: the presence of the correct health warning and the correct tax stamp identified a legal pack, whereas the absence of either of these features identified an illegal pack. In TPackSS countries that either did not use tax stamps (China, the Philippines and Mexico) or did not apply them on all packs at the time of data collection (Egypt), the legal/illegal status was determined based entirely on the health warning.

The price per pack was normalised to 20 sticks per pack and the local currency was converted to US$ using XE.com using the conversion rate on the date of purchase. In order to account for the non-normal distribution of cigarette prices, we employed geometric means to compare prices across cigarette types (eg, legal, illegal, Cheap Whites, non-Cheap Whites) within a specific country.

The main source of information on the brand ownership of TPackSS packs was the online trademark registries managed by the European Trade Mark and Design Network and by the World Intellectual Property Organization.^34^ ^35^ If the brand owner was not identified using these sources, we conducted a general Google search using information from TPackSS such as the brand name, the country of origin and the name of the manufacturer available on most packs. We cross-verified information obtained via a Google search using at least two different sources of information. In cases of multiple brand ownership, we recorded all brand owners associated with the particular brand.

Given the bias of TTCs in evaluating the Cheap Whites phenomenon, we analysed the data from the non-industry and the industry sources separately. We refer to the original source of the information independent of who disseminated it.

## Results

We found the term ‘Cheap/Illicit Whites’ first being used in the WCO 2009 report,^13^ while two later sources reported that the term was created by the tobacco industry.^3^ ^21^ Since 2009, many government institutions, international organisations and TTCs have come up with their own definitions of the phenomenon. Online supplementary appendix 1 provides an overview of these definitions and how they evolved over time. Online supplementary appendix 2 describes the occurrence of Cheap White brands on the market and their growing presence in the illicit cigarette supply.

Available data sources identified 82 Cheap White brands with more than one-third (31 brands) confirmed by multiple sources and 51 brands reported only by TTCs (see online supplementary appendix 3).

We found the names and locations of 53 Cheap White manufacturers who operate at least 82 production facilities (see online supplementary appendix 3). We identified the geographical location of manufacturing facilities for an additional nine brands, but we did not find their manufacturers’ names. A manufacturer can have multiple production facilities in multiple countries. For example, Baltic Tobacco, who produces Jin Ling, has 19 factories located in Russia alone, in addition to factories in Ukraine and Moldova. Some of these factories may be acting as a franchise, but the Jin Ling brand is also licensed to other manufacturers.

Among the production facilities with a known manufacturer, 12 (15%) were located in the EU, and 32 (39%) were located in Europe but outside the EU. Three manufacturers were located in the UAE, but this is likely to be an underestimation given that the names of manufacturers for another seven brands that are also manufactured in the UAE are unknown.

One-third (n=27) of the Cheap White production facilities were in free zones of Russia, Cyprus and the UAE. Free zones, a class of special economic zone designated by the trade and commerce administrations of various countries, are known to facilitate the production and distribution of Cheap Whites as they are subject to weak regulations.^22^ This finding is consistent with the information provided by the UK's TMA,^31^ Her Majesty's Revenue and Customs (HMRC)^24^ and the EC.^18^

We found 81 different trademark owners located in 36 countries (see online supplementary appendix 3). China is home to 11 trademark owners. The UK and the UAE host seven and six trade mark owners, respectively. The brand owner and the manufacturer are not necessarily the same entity, and a brand can have both multiple manufacturers and multiple trademark owners. The trademark ownership of Cheap White brands is very complex with multiple owners dividing the global market into geographical segments, registering different features of a brand (name, pack design, etc), and sometimes even retrospectively changing trademark registry (eg, Jin Ling trade mark).^36^ A brand can have different features registered with different companies. For example, ‘Yes’ brand has as many as four different pack designs, all registered with different companies. TTCs also own some of the trademarks, in many instances using names of less-known companies they own—for example, the trademark of Premier in Peru is owned by Tabacalera Nacional S.A.A, which belongs to BAT; it is also owned by LLC Petro in Russia (owned by JTI) and by Abal Hermanos. S.A. in Uruguay (owned by PMI). Generally, TTCs’ brand registration is geographically more comprehensive compared with Cheap Whites.

TPackSS contains information on 37 Cheap White brands. Among them, 25 brands (67.5%) had neither the correct health warning nor the required tax stamp at least in one country of purchase and 15 brands (40.5%) had neither the correct health warning nor the required tax stamp in any of the countries of purchase. There were 13 Cheap White brands (35%) that complied with both tax stamp and health warning requirements in all TPackSS countries. In 4 of the 14 countries (Russia, Brazil, Indonesia and Mexico), all Cheap White brands had the proper health warning and tax stamp. The major source countries of Cheap Whites, Russia and Ukraine, have the majority of Cheap White brands available legally on their domestic markets: only 4 of the 45 packs of Cheap White brands purchased in the Ukraine were illicit (none of the 47 packs in Russia). On the other hand, the majority of Cheap White packs purchased in Bangladesh (86%), Pakistan (94%), Thailand (72%), the Philippines (80%), and Viet Nam (93%) were not compliant.

A specific Cheap White brand is generally sold in a country either legally or illegally, with the exception of three brands—Esse, YunYan and Vigor. TPackSS found that Esse were sold both legally and illegally in China (legally in Beijing; illegally in Shanghai and Guangzhou), in Thailand (legally in Chiang Mai; illegally in Bangkok), in the Philippines (legally in Cebu City; illegally in Cebu City and Manila) and in Turkey (legally in Istanbul and Konya; illegally in Diyarbakir). Twenty packs of YunYan were sold legally in Beijing, Shanghai, Kumming and Chengdu while one pack was sold illegally in Guangzhou. One of the six packs of Vigor sold in Turkey was illegal. Illegal Vigor and Esse were significantly cheaper than their legal counterparts in Turkey and the Philippines, while there was no price difference between the legal and illegal Esse and YunYan in China and Thailand. In total, 86% of the 132 illegal Cheap Whites packs in the TPackSS database were not purchased from large retailers such as superstores, supermarkets and grocery stores.

Multiple sources state that Cheap Whites are generally sold at half the price of domestic duty paid cigarettes.^2^ ^37^ For example, illegal Cheap Whites in the UK were sold for £2.50 in 2014, compared with an average price of £5.50–£6.50 for legal cigarettes.^28^ ^31^ However, there are also reports that illegal Cheap White brands from Belarus such as Fest, Minsk and Nz were sold for as little as €0.2 in some EU markets.^38^

The TPackSS database allowed us to compare average prices of Cheap Whites and non-Cheap Whites in 14 countries according to their legal status. Of a possible 28 pairs, the comparison generated nine statistically significant results (p<0.05). Among both legal and illegal cigarettes, Cheap Whites were cheaper compared with other brands in the same legal/illegal category with the exception of India where legal Cheap Whites were on average more expensive than legal non-Cheap White brands. Excluding India and considering only the remaining eight statistically significant results, Cheap White brands were on average 29% less expensive.

The comparison of legal and illegal Cheap Whites prices produced mixed results: in three of the five countries where the results were statistically significant, the illegal Cheap Whites were more expensive than their legal counterparts.

Surprisingly, the average prices of legal non-Cheap White brands were lower than the average prices of illegal Cheap Whites in five of the seven countries where the results were statistically significant. With the exception of Turkey, the cheapest Cheap White cigarettes purchased in any TPackSS country irrespective of their legal status were still more expensive than the least expensive legal non-Cheap White brand.

## Discussion

We found that Cheap White brands and TTC brands have many similarities. They are sold legally and illegally and their production facilities are located in multiple countries with the manufacturer and the trademark owner sometimes being different legal entities. On average, the prices of Cheap Whites were lower compared with other brands, but the price differences were not as large as reported anecdotally from Western Europe. In 13 of the 14 TPackSS countries, the least expensive legal brand was cheaper than the least expensive Cheap White brand.

The trademark registration of Cheap White brands is less comprehensive compared with TTC brands as it is geographically limited.

Both enforcement agencies and TTCs report an upward trend in the market presence of illegal Cheap Whites. In order to penetrate new markets, cigarettes are sold without payment of all applicable taxes. This approach was employed by TTCs when they used illicit routes to secure their access to new markets in Asia, Africa and the former Soviet Union countries.^3^ The manufacturers of Cheap Whites seem to apply the same strategy. This would seem to explain why TTCs try to draw attention to Cheap Whites—they oppose them not because they are illegal, but because they represent competition. In addition, TTCs seem to use the presence of illegal Cheap Whites to divert attention from their own contribution to the illegal cigarette market.^10^ ^39^ ^40^

Despite the attention paid to Cheap Whites by the TTCs and the media, the majority of illicit cigarettes consumed in Europe, for example, still consist of TTC brands.^10^ ^41^ This means that illicit cigarette trade is not likely to disappear by eliminating Cheap Whites but rather by focusing on illicit trade of all tobacco products. The WHO Protocol to Eliminate Illicit Trade in Tobacco Products requires Parties to the Protocol to take measures to effectively control the supply chain of tobacco products.^42^ Ratification of, or accession to, the Protocol by the majority of countries, and especially by countries where Cheap Whites are manufactured, would be a step in the right direction. By the time this happens, however, some Cheap White brands might already be well established in some markets and able to compete with other TTC brands even if taxes on them are fully paid.

### Limitations

Our study has several limitations. First, the majority of data on Cheap White brands are from Europe, because the seizures there are better documented compared with other regions. Second, our results with respect to legal or illegal status of a particular brand and its market price are based only on data from 14 TPackSS countries and none of these countries are located in the EU. This limits our ability to assess the legal status of Cheap Whites in the EU, where the majority of them are seized. Nevertheless, we found that even outside the EU, two-thirds of Cheap White brands were sold illegally, primarily in the countries where Cheap Whites are not produced. Third, our definition of legal and illicit products is rather broad, given that we could not verify compliance with other legal requirements such as tar, nicotine and carbon monoxide emissions, for example. Fourth, our online searches were limited to sources in English and Russian. Even though we have reached out to our colleagues in Latin America to cross-verify our results, we most likely missed data published in other languages.

## Conclusions

On the basis of our findings, we challenge the notion of Cheap Whites being only illegal products that deprive governments of tax revenue. However, many Cheap Whites appear to evade some taxes, thus undermining tobacco tax policy. Moreover, the availability of fully taxed cheap cigarettes is a public health concern given their high affordability.

**What this paper adds**

- The market presence of Cheap White cigarettes is increasing.
- Tobacco companies are pointing to Cheap Whites as the main source of illicit cigarettes.
- The Cheap White phenomenon has remained unexplored in the academic literature.
- We present an up-to-date list of Cheap White brands, and identify most of their manufacturers and trademark owners.
- Contrary to the general notion that all Cheap Whites are illegal, we identified markets where some Cheap White brands are sold legally.
- Cheap Whites should not distract policymakers from addressing illicit trade in all tobacco products as is proposed by the WHO Protocol to Eliminate Illicit Trade in Tobacco Products.

## Supplementary Material

Web supplement
