# Supplementary material for: A closer look at ‘Cheap White’ cigarettes
Source: Tob Control. 2015 Sep 28;25(5):527–31. doi: 10.1136/tobaccocontrol-2015-052540 (PMC5036225; doi:10.1136/tobaccocontrol-2015-052540)
Supplement: Web supplement [file tobaccocontrol-2015-052540-s1.pdf]

Appendix 1:

Definitions of Cheap Whites from non-industry sources

(Text underlined to help the analysis)

Even though the term "Cheap Whites" appeared in the 2009 WCO report,[13] it was first defined in 2011 by the European Commission (EC) and Europol. The EC defined “Illicit White” as “brands manufactured legitimately in one market, either taxed for local consumption or untaxed for export, and sold knowingly to traders who transport them to another country where the products are sold illegally without domestic duty paid.”[19] Europol's definition was strikingly different: they defined "Cheap Whites" as "the tobacco industry’s term for cigarettes produced entirely independently of the traditional tobacco manufacturers. According to Europol, “Cheap Whites” are cheap cigarette brands, often of reasonable and consistent quality, that provide a good alternative to counterfeits, whose quality can vary significantly.[21]

Given that the 2011 EC definition implies that TTCs brands in illicit trade channels are Cheap Whites, an update was made in the 2013 EC document, which narrowed the definition and brought it closer to Europol's definition making clear that “Cheap Whites” are "other brands", i.e. “…cigarettes produced entirely independently of the traditional tobacco manufacturers”.[18] This was rather surprising given that one of the most commonly seized Cheap White brands in Europe (Classic) was manufactured by one of the TTCs (Imperial Tobacco).[6 33]

A definition similar to the first EC definition has been adopted by another EU institution – Office de Lutte Anti-Fraude (OLAF): “Cheap Whites are cigarettes manufactured legitimately in one country where they may have a domestic market or may not have a market

at all in the EU, but are smuggled into or sold illegally in another country/market, where they do not have the license to be sold legally." [13]

The World Customs Organization defines Cheap Whites as cigarettes with "legal production in the source country and the usually legal sale to the first purchaser" while "the overall cigarette consumption at national level does not correspond to the volume of production.[2] A large number of 'Cheap Whites' cigarettes are not legally available in the destination countries and generally do not comply with regulatory standards". WCO also defines a sub-category of Cheap Whites - product doubles - that feature graphics and colors that are very similar to well-known brands, but have different, often similar names.[2]

The definitions disseminated by OLAF and the original 2011 EC definition imply that Cheap Whites can be legitimate in countries where they are produced, but not in other markets, while the WCO definition opens the possibility that some Cheap Whites are sold legally in the destination country.

On the other hand, the definition adopted by the UK government, and also the only definition available in the academic literature, implies that Cheap Whites have no legitimate market anywhere. The UK's HMRC defines Cheap Whites as "brands that are legally manufactured by companies, often based in emerging economies, with the intent on exporting illegally to other countries through a smuggling network. The products are then sold illegally without domestic duty being paid".[1] Gilmore et al. (2014)[10] also define Cheap Whites as non-TTC branded cigarettes that are legally produced but have no legitimate market.

In 2014, Interpol introduced a new feature to the definition when it specifically mentioned trademark registration: "New cigarette brands (generally with registered trade brands) produced legally in one jurisdiction but produced intentionally for smuggling into other

countries where there is no prior legal market for them. Tax possibly paid in production country, but rarely. Products are lawfully exported from the production country and tax will not be paid in the destination country."[22]

To summarize, various institutions define Cheap Whites differently, which may reflect the varying aspects of illicit trade that they focus on. The government agencies stress tax evasion while the enforcement agencies focus on the legal aspect of the trade. The definitions of Cheap Whites are often confusing and change over time, which could reflect the evolving nature of the industry including the business model under which it operates.

Definitions of Cheap Whites by TTCs

The majority of the industry sources use the term "Illicit Whites" instead of "Cheap Whites", because it better suits the industry rhetoric about the issue. We found the first TTCs definition of Illicit Whites in the 2011 Project Star (PS) report funded by PMI[29] defining them as brands that are mainly produced for the purpose of smuggling. The 2011 PS report categorizes all non-TTCs brands found in the EU without correct marking (e.g. local tax stamps) or Duty Free labelling as Illicit Whites. The subsequent 2012 PS report[6] published in 2013 paid much more attention to the definition of Illicit Whites: "Cigarette brands manufactured outside the EU and/or in Free Trade Zones, and distributed in such a way that a large share of their volume is sold at a very low price and consumed in EU countries, although these brands are in general not registered there and not destined to be sold at legitimate retail in such markets". The 2012 PS report also defines Domestic Whites as domestic cigarette brands that are priced below the minimum tax rate.[6]

The PS definitions imply that Cheap Whites can have legitimate markets even in countries where they are not manufactured. The geographical location of Cheap White production, low

prices, market registration and distribution via illegal distribution channels were new features of the PS 2012 definition that clearly distinguish Cheap Whites from TTCs brands.

Interestingly, the Asia-11[4] report published in the same year as the 2012 PS report and also funded by PMI defines Illicit Whites a bit differently: “Cigarettes that are usually produced legally in one country/market, primarily for smuggling. While they may also be exported legally from some countries, they are smuggled ….. to point of sale where they have no legal distribution and are sold without payment of tax”. This definition implies legitimacy of Cheap Whites in some countries, but clearly excludes brands produced by TTCs because these are not primarily produced for the purpose of smuggling (even though some are produced for this purpose[10]) and do not have legal distribution. The follow-up report from Asia published in 2014[30] does not discuss the issue of Illicit Whites at all.

The common features of the TTCs definition are: a clear distinction between the Cheap White and the TTCs brands in terms of their legal status/distribution and a focus on low price or no tax payment, which creates “unfair” competition for TTCs since they cannot compete with the low prices of Cheap Whites. The definitions used by Europol, the 2013 EC and the TTCs are fairly similar.

All definitions, independent of the source, highlight that Cheap Whites are manufactured legally, but a lot of them are sold illegally. It seems that the only difference between the Cheap White and TTCs brands is the disproportionate share of the legal and illegal sales, and the competitive pricing/tax advantage of Cheap White brands stressed by the TTCs.

Appendix 2:

Market share of illegal Cheap Whites

One possible way to estimate the share of the illicit cigarette market composed of Cheap White brands is to analyze seizure data. The first seizures of Jin Ling, a prominent Cheap White brand, were reported in Europe in 2005.[11] The following year, Jin Ling became one of the most frequently seized cigarette brands in the EU.[22] By 2007, the UK authority reported a growing share of Cheap White brands among all seized cigarettes.[24] Shortly after Cheap Whites appeared in Europe, their seizures were reported in Asia[3] (in 2008) and Africa[23] (in 2000) where they were also commanding a growing share of the illicit market. The tobacco industry reports that there are at least six factories in Zimbabwe that produce over 20 brands of Cheap Whites, many destined for South Africa.[32]

In 2010, the WCO signaled “A significant and growing threat from cheap white cigarettes”[14] as their presence spread to many European countries.[13] The 2011 WCO report states that “the popularity of “Cheap Whites” cigarettes is increasing at the expense of counterfeit cigarettes”,[15] while Jin Ling became one of the main smuggled brands in Europe.[4] According to the European Commission, Cheap Whites accounted for approximately 20% of seizures in the EU in 2011,[17] but already 26% a year later.[41] WCO reported even faster growth of the number of seizures of the Cheap White brands (52% increase) and rising quantities of Cheap Whites seized (17% increase) during the same period.[2] Most of these cigarettes were seized in Europe, although this might be the product of better enforcement and reporting practices in that region. By 2012–13 the majority of large seizures in the UK were of Cheap Whites.[1] A 2013 report by Irish customs shows 76% of seized cigarettes in 2012 were Cheap Whites, up from 46% in 2011.[27]

Interpol estimates that Paraguay manufactures more than 2600 cheap cigarette brands, where the majority are not sold in the domestic market.[22] Cheap Whites produced in Paraguay have been seized across Latin America.[32]

The TTCs have been stressing the growing presence of Cheap Whites since the early 2010s as a way to turn public attention away from their brands as part of the illicit trade channels.[41] According to the TTCs, the consumption of Cheap White brands increased by 3% just between 2011 and 2012, reaching 16 billion sticks (or 24% of illicit cigarette market) in the EU alone.

Based on their ongoing collection of discarded packs on the streets of major European metropolis, the TTCs were able to detect a shift in the supply of Cheap Whites: Cheap White brands occupying the largest share of the illegal cigarette market in Europe until 2011 (Classic, Jin Ling and American Legend) began to lose their market share to new emerging Cheap White brands such as NZ, Fest, Premier, and Minsk, primarily coming from Belarus.[6] This trend has been confirmed by illegal cigarettes seizures by the Customs Departments in Poland[25] and the Baltic States.[41]

Appendix 3: List of Cheap White cigarettes

**Brand**	**Manufacturer/s (manufacturing site)**	**Trademark owner/s (headquarter)**
Brands identified as Cheap Whites by multiple sources		
821	Manifattura Italiana Tabacco (Italy)	Manifattura Italiana Tabacco (Italy)
Business Club	European Tobacco (Lebanon)	Vian Ltd (Bulgaria)
Capital	• Vietnam National Tobacco Corp. (Vietnam)<br>• Oriental Group (UAE)	• Vietnam National Tobacco Corp. (Vietnam)<br>• Oriental Group/Anthony D'Souza (UAE)<br>• Medi Plus Tec Medizinisch-Technische (Germany)
Classic	Imperial Tobacco (India, Ukraine, Russia)	Imperial Tobacco (UK)
em@il	Manifattura Italiana Tabacco (Italy)	Manifattura Italiana Tabacco (Italy)
Fu-qi (Good Fortune)	Qu Jing Cigarette Factory (China)	Qu Jing Cigarette Factory (China)
George Karelias and Sons	Karelia Tobacco Company Inc. (Greece)	Karelia Tobacco Company Inc. (Greece)
Gold Classic	Explosal Ltd (Cyprus and possibly other countries)	Explosal Ltd (Cyprus)
Golden Bridge	• Xiamen Cigarette Factory (Fujian: China)<br>• Fujian Industrial Co. (Fujian: China)	• China National Tobacco Corporation (China)<br>• China Tobacco Fujian Industrial Co., Ltd. (China)
Half Time 5	Selangor Cigarette Factory (Malaysia)	Natuzi Trading Pte Ltd (Singapore)
Hatamen	• China Tobacco Shandong Industrial Corporation (China)<br>• (UAE)	China Shandong Industrial Corporation (China)
Jet	Sumatra Tobacco Trade Company (Indonesia)	Sumatra Tobacco Trade Company (Indonesia)
Hero	Sumatra Tobacco Trade Company (Indonesia)	• Sumatra Tobacco Trade Company (Indonesia)<br>• Philip Morris (China)
Jim	(UAE)	Mediterranean Star Llc (UAE)
Jin Ling	• Baltic Tobacco (19 locations in Russia, 1 Ukraine, 1 Moldova)<br>• BE Tobacco Company Khamaday (Donetsk: Ukraine)<br>• (Chisinau: Moldova)	• MegaEvrobud Limited Liability Co. (Ukraine)<br>• Bluestar Worldwide Logistic Co. (Philippines)<br>• Baltic Tobacco (Kaliningrad, Russia)<br>• Francisco Javier Sarmiento Santana (Spain)<br>• Ariki Pte Ltd (Singapore)<br>• Ilcenco Vsevolod (Moldova)
June Slims	• GTC Industries Ltd (India)	-
Luffman	PT Leadon International (Indonesia)	Leadon Tobacco International Pte Ltd (Singapore)

Manchester	Stolichnaya Tobacco Factory (Moscow: Russia)	• J.S.S Tobacco Ltd (UK)<br>• Jamal Abdul Nasser Mahmood Al Mahamid (UAE)<br>• Manchester Cigarette Co (UK)
Master	(UAE)	Dunhill Tobacco Of London Limited (UK)
Mond	• Gulbahar Tobacco International FZE (previously Concord Tobacco International Corp.) (Dubai: UAE)	• Mr. Kamal Kareem Ahmad (UAE)<br>• Gulbahar Tobacco International FZE
Mond International	• Gulbahar Tobacco International FZE (previously Concord Tobacco International Corp.) (Dubai: UAE)	• Mr. Kamal Kareem Ahmad (UAE)<br>• Gulbahar Tobacco International FZE
Mo-shen	-	-
NZ	Grodno Tobacco Factory Neman (Belarus)	• Grodno Tobacco Factory Neman<br>• Otkrytoe aktsionernoe obshchestvo (Belarus)
Palace	PMI (Brazil and possibly other countries)	• PMI (USA)<br>• ESPL Marketing Pte Ltd (Singapore)<br>• Century Tobacco (UAE)
Raquel	Explosal Ltd (Cyprus and possibly other countries)	Explosal Ltd (Cyprus)
Regal	-	Imperial Tobacco (UK)
Richman	(UAE)	Stepanchuk Ruslan Dmytrovych (Ukraine)
Shan	-	-
Top Mountain	(UAE)	-
Vigor	European Tobacco (Lebanon)	European Tobacco (Turkey)
Yesmoke	Yesmoke Tobacco Factory (Italy)	Yesmoke Tobacco S.P.A (Italy)
Brands identified as Cheap Whites only by industry		
American Legend	Karelia Tobacco Company Inc. (Greece)	Karelia Tobacco Company Inc. (Greece)
Austin	-	Landewyck Tobacco S.A. (Luxembourg)
Bayron	Baltic Tobacco Factory (Russia)	Baltic Tobacco Factory
Bon	-	-
Bon International	(UAE)	-
Bredal	-	-
Broadway	Montepaz (Uruguay)	• Compania Industrial de Tabacos Monte-Paz Uruguay<br>• Dubek Ltd. (Israel)<br>• Philip Morris (Australia, China)<br>• Imperial Tobacco (Canada)
Business Man	-	-
CK	-	-
Compliment	Baltic Tobacco Factory (Russia)	-
Corsair	King's Tobacco (Bulgaria)	Ednolitchno Drujestvo S Ogranitchena Otgovornost (Bulgaria)

Derby	• British American Tobacco (Bangladesh)<br>• Souza Cruz (Brazil)	• British American Tobacco (Australia, UK)<br>• China Tobacco Anhui Industrial Co., Ltd. (China)<br>• D. Ritchie & Co. (Canada)
Ducal	Heintz Van Landewyck (Trier: Germany)	• Heintz Van Landewyck (Luxembourg)<br>• Bluestar Worldwide Logistic Co. (Philippines)
EB	• Baltic Tobacco Factory (Lvov: Ukraine,<br>Kaliningrad Zone Free: Russia)	• Bugatti International S.A. (Luxembourg);<br>• MegaEvrobud Limited Liability Co. (Ukraine)
Elixyr	Heintz Van Landewyck (Trier: Germany)	Heintz Van Landewyck (Luxembourg)
Esse	KT&G (Korea)	KT&G Corporation (Korea)
Fest	Grodno Tobacco Factory Neman (Belarus)	Grodno Tobacco Factory Neman (Belarus)
GB	-	Xiamen Tobacco Industrial Co., Ltd. (China)
Goal	-	-
Gold Mount	• London Tobacco (overseas) Ltd (UK)<br>• Kaane American International Tabacco (UAE)	Kaane American International Tobacco Co. Ltd. (UAE)
Golden Seagiies	-	-
GR	-	-
Hd	European Tobacco (Turkey)	British American Tobacco (UK)
Karelia	Karelia Tobacco Company Inc. (Greece)	Karelia Tobacco Company, Inc. (Greece)
Kiss	BAT Donskoj tabak (Russia)	Innovation Tobacco Company Ltd (UK)
Lifa	Baltic Tobacco Factory (Russia)	-
Magnat	Grodno Tobacco Factory Neman (Belarus)	Grodno Tobacco Factory Neman (Belarus)
Marble	(UAE)	Hongta Tobacco (Group) Co., Ltd. (China)
MG	-	Dubek Ltd (Israel)
Minsk	Grodno Tobacco Factory Neman (Belarus)	Grodno Tobacco Factory Neman (Belarus)
Modeng	-	-
More	• JTI (Ukraine, Russia)<br>• PMI (Philippines)	JTI (Japan)
My Way	-	-
Napoli	Collezione Italiana Tabacchi (Italy)	Collezione Italiana Tabacchi (Italy)
Nelson	• Dubek Ltd (Israel)<br>• Israel Cigarette Company Ltd. (Is rael)	• The Israel Cigarette Co. Ltd. (Israel)<br>• Dubek Ltd. (Israel)<br>• Senior Service (Overseas) Limited (UK)<br>• Manufacture De Tabacs (Luxembourg)<br>• Lidl Stiftung & Co. Kg (Germany)<br>• Selected Tobacco S.A. (Costa Rica)

		• Melfinco S. A. (Liechtenstein)
Paladium	• Veneto Tabacos (Paraguay)<br>• Espert S.A. Tabacalera Argentina (Argentina)	• Veneto Tabacos (Paraguay)<br>• Fab. de Cigarros Paulista Gracioli (Brazil)
Penang	-	Hanchen Tobacco (Europe) B.V. (Netherlands)
Portman	Melfinco (Greece)	Melfinco S.A. (Liechtenstein)
Premier	• Grodno Tobacco Factory Neman (Belarus)<br>• Protabaco S.A. (Colombia)<br>• Abal Hnos. S.A. (Uruguay)<br>• Tabacalera Nacional S.A. (Panama)<br>• LLC Petro (Russia)	• Grodno Tobacco Factory Neman (Belarus)<br>• British American Tobacco Australia Limited (Australia)<br>• Van Nelle Canada Limited (Canada)<br>• Japan Tobacco Inc. (Japan)<br>• Renwick Of Canada Inc.- Renwick Du Canada Inc. (Canada)<br>•Protabaco S.A. (Colombia)<br>• Abal Hnos. S.A. (Uruguay)<br>• Tabacalera Nacional S.A. (Panama)<br>• LLC Petro (Russia)
President	• PMI (Ukraine)<br>• European Tobacco (Lebanon)<br>• International Cigarette Industries (Pvt.) Ltd. (Pakistan)	• PMI (USA)<br>• European Tobacco (Turkey)<br>• Ohserase Holdings, LLC (U.S.)<br>• Imperial Tobacco (UK)<br>• International Cigarette Industries (Pvt.) Ltd. (Pakistan)
Queen	Grodno Tobacco Factory Neman (Belarus)	• Fantasia Distribution, Inc. (Canada)<br>• Grodno Tobacco Factory Neman (Belarus)
RGD (Red Golden Dragon)	• Hubei Industrial Co. Ltd. (China)<br>• Wuhan Cigarette Factory (China)<br>• PMI (Poland, Czech Republic, Hungary)	• China Tobacco Hubei Industrial Co., Ltd. (China)<br>• Wuhan Tobacco Group (China)
Rodeo	• Tabesa (Paraguay)<br>• (Mexico)<br>• Coimexpor Argentina S.A (Argentina)	• Natuzi Trading Pte Ltd (Singapore)<br>• Coimexpor Argentina S.A (Argentina)
Ronhill	Tobacco Factory Rovinj (Croatia)	Tobacco Factory Rovinj (Croatia)
Super Sunday	-	-
Toro	-	-
VIP	Grodno Tobacco Factory Neman (Belarus)	• International Masis Tabak (Armenia)<br>• European Tobacco (Turkey)<br>• Cyf Enterprise Limited (Singapore)<br>• Grodno Tobacco Factory Neman (Belarus)
VP international	-	-
Walton	-	Ray Tobacco Inc. Llc. (USA)

Yes	• Mastermind (South Africa)<br>• ITABA (Brazil)<br>• La Soberana (Paraguay)<br>• Taiwan Tobacco & Wine Board (Taiwan)	• Potomac Tobacco Company Ltd. (Belgium, British Virgin Islands)<br>• ITABA (Brazil)<br>• Taiwan Tobacco & Wine Board (Brazil)
Yun Yan	Hongyunhonhge Tobacco Co., Ltd (China)	• Hongyunhonhge Tobacco Co. (China)<br>• P.T. Permona (Indonesia)
